# Useful life lessons for health and well-being: adults’ reflections of childhood experiences illuminate the phenomenon of the inner child

**DOI:** 10.1080/17482631.2018.1441592

**Published:** 2018-02-28

**Authors:** Margareta Sjöblom, Kerstin Öhrling, Catrine Kostenius

**Affiliations:** ^a^ Department of Health Sciences, Luleå University of Technology, Luleå, Sweden

**Keywords:** Adulthood, health literacy, health promotion, mental health, existential aspects, social interaction

## Abstract

**Purpose:** The aim of this study was to describe and gain more knowledge about the phenomenon of the inner child in relation to health and well-being reflected in events during childhood experienced by adults. **Method:** In this hermeneutical phenomenological study, 20 adults, 10 men and 10 women aged 22–68, were interviewed. **Results:** The analysis of the data illuminated the phenomenon of the inner child in one theme: *Gaining useful life lessons through childhood experiences*, made up by four sub-themes: *Sharing relationships*, *playing to heal*, *being strong or frail* and *supporting the next generation*. **Conclusion:** The participants’ experiences of events during childhood were illuminating the phenomenon of the inner child as promoting or hindering health and well-being and impact human adaptation throughout life. Our findings indicate that the participants learned useful life lessons suggesting that experiences during childhood can help us to adapt across the life span and over generations, and this is the essence of the inner child. Our findings also contribute to the health literacy discussion and detail how knowledge and action competency is developed in mental, social and existential dimensions of health and well-being.

## Introduction

According to the Public Health Agency of Sweden (2016), many health problems have decreased in recent decades. The average life expectancy continues to increase, which is a result primarily of the significant decrease in mortality rates of cardiovascular diseases and cancer. However, the National Board of Health and Welfare (2003) concluded that stress-related problems like mental illness and aches seem to be escalating. Barry (2009) identified determinants across life, which promoted positive mental health at the structural, community and individual levels. She argued that mental health promotion has a key role in creating well-being by empowering individuals and communities positively by reorienting public policies and services across society.

Adults today live in a stressful changeable world that creates challenges in coping with health and well-being (Eriksson, 2016). People who experience their working situation as stressful and mentally exhausting most often have low educational and poor socio-economic background, according to Norlund et al. (2010). They explain that these factors are more strongly related to stress than to gender differences. Stress can be connected to burnout when stress is not managed, i.e., an overwhelming workload with lack of control, appreciation and support and little possibility to recover (Maslach & Leiter, 1997). According to Theorell and Karasek (1996), the social dimension, including support from colleagues and friends, can be viewed as a health-promoting factor as social support reduces stress and increases control over the work situation. Health promotion is a way for people to increase control over their health and goes beyond being a resource for everyday life to reach a state of complete physical, mental and social well-being (WHO, 1986, 1998a).

During the last few decades, research on health-related quality of life has emphasized the importance of existential health including, for example, aspects like meaning and purpose in life, experience of awe and wonder, inner peace, hope optimism and faith—sometimes referred to as spiritual health (Melder, 2011). Fetro (2010) included “physical, emotional, mental, social, and spiritual” dimensions of health when discussing opportunities to promote health literacy (p. 258). The World Health Organization’s definition of health literacy is “the cognitive and social skills and ability of individuals to gain access and to use information and understand ways which maintain and promote good health” (WHO, 1998b, p. 10). Mancuso (2011) suggests tools to overcome health literacy barriers to integrate beliefs, values, social and cultural traditions with information related to health. This changes health-related behaviour based on the individuals’ view of the world and makes informed consent. In addition, Kickbusch, Wait, and Maag (2005) highlighted the action competency aspect of health literacy as making sound health decisions in the context of everyday life in addition to finding and making sense of health information. According to Nutbeam (2008), health literacy can be considered as a risk factor or as an asset. He concluded that “both conceptualizations are important and are helping to stimulate a more sophisticated understanding of the process of health communication in both clinical and community settings, as well as highlighting factors impacting on its effectiveness” (p. 2072). Similar to viewing health literacy as an asset, Ringsberg, Olander, and Tillgren (2014) explained that it can be looked upon as a competence useful in everyday life to promote and maintain good health across a lifetime. According to Firman and Russel (1994), human beings’ life journey includes all the past hidden ages that impact human lives—this is a phenomenon they describe as the inner child. The phenomenon of the inner child can be compared to Jung’s divine child—he argued that this is an extension of the individual to the life of mankind (Jung & Kerényi, 1969[1963]). According to Weston (2009), the individual brings certain strengths and knowledge into adulthood that can be considered as a contribution of the inner child. Similarly, Assagioli (1973) argued that the psychosynthesis of the ages keeps the best aspects of every stage of development from our past alive in the present.

## Aim

The aim of this study was to describe and gain more knowledge about the phenomenon of the inner child in relation to health and well-being reflected in events during childhood experienced by adults.

## Method

The frame of reference in this study was inspired by van Manen’s (1990) hermeneutical phenomenological approach to researching lived experience, which was based on collecting and analysing human beings’ stories to understand a phenomenon. The phenomenon in focus is the inner child in relation to health and well-being as reflected in events during childhood experienced by adults. According to van Manen (1990), the analysis process includes three steps: seeking meaning, thematic analysis, and interpretations with reflections. This is a systematic attempt to uncover and describe the structure of the life-world as we immediately experience it.

### Participants and procedure

The participants were adults recruited through a primary school part of a larger project where children also were invited to be interviewed; results from the study with the children will be presented in a forthcoming article. All the authors participated in one meeting with the staff in the primary school and in another meeting with parents in the primary school. Twenty people participated in each meeting, totalling 40 people. Twenty people agreed to participate, 10 men and 10 women. The ages of the 10 men ranged from 22 to 68 years and the ages of the 10 women ranged from 29 to 52 years. Five of the men were parents to children in the primary school and five were part of the school staff (one principle, three teachers and one custodian). Seven of the 10 men had children of their own. Nine of the women were parents to children in the primary school and one woman was a teacher. All the women had children of their own. Each participant was interviewed once during March to May 2016. The participants received oral and written information during the meeting with the three authors at the school. The first author interviewed 19 participants on the premises of the school, and one participant was interviewed at their own home according to the participant´s wishes.

### Data collection

Data was collected with open-ended interviews conducted by the first author aiming at capturing the participants’ essential human experiences as described by van Manen (1990). A pilot interview was conducted to test the research questions. This resulted in fewer questions. Instead of many detailed questions, the interviews started with the following comprehensive question: *Please describe significant events from your childhood that you have carried with you throughout your life.* This approach gave the participants flexibility to choose which experiences to share. Supporting questions were asked including: *Is there anything in what you have narrated that has affected your health and how you feel today? Is there anything in what you have narrated that you have forwarded to your children?* The interviews lasted between one and one-and-a-half hours and were tape-recorded and transcribed verbatim by the first author. The findings include quotes followed by a code indicating which participant spoke and the person’s gender: A1 to A10 for women and B1 to B10 for the men.

### Data analysis

A hermeneutic-phenomenological data analysis was used following van Manen’s (1990) ideas on capturing lived experience to develop a richer and deeper understanding of a human phenomenon. All authors were involved in this process. According to van Manen (1990), seeking meaning on different levels creates a holistic approach. The first step—seeking meaning—focused on finding the significance in each interview, while also capturing the meaning of the complete picture, including all the interviews. The analysis started by all the authors reading the transcribed interviews multiple times, to get an overall meaning of the phenomenon. Each interview was read to find phrases or expressions that captured the essence of the phenomenon of the inner child in relation to health and well-being. In this first step, the analysis implied a back-and-forth movement between the whole and the parts inspired by van Manen (1990). The second step of the analysis—thematic analysis—focused on gripping the experiential structures that made up the participant’s experiences. In this phase the text was analysed in detail with focus on every sentence, to find the similarities and differences in the adults’ lived experiences. The identification of themes was made by all authors separately, and they were thereafter discussed and formulated together. Units of text from the interviews were organized in several steps into different experiences. At the end, these were reduced to one theme with four sub-themes to capture the participant’s lived experiences. The third step—interpretation with reflection—concluded the analysing process described by van Manen (1990), as a process recovering the embodied meaning of the text in an insightful and free way, trying to answer the question “what is it like?” (p. 46). This was an effort to capture a deeper understanding of the phenomenon to be communicated in the themes. With these in mind, all the authors discussed the themes and reached consensus about their formulation. The process of writing and rewriting was also a part of the analysis and continuously discussed among the authors.

### Ethical considerations

The participants were given oral and written information about the study as outlined in the Helsinki Declaration. In accordance with Swedish ethical law (SFS, 2003:460), informed consent was collected from the participants and participation was voluntary. The participants received information about their autonomy, confidentiality and their role in the project. The participants had the option to withdraw from the project at any time for any reason. They were informed that no one except the authors of this article would have access to the collected material. All interviews were coded to protect the identities of the participants. The code list will be destroyed after the analysis is finished and the manuscript is published. No names, addresses or identification numbers were collected. All through the analysis process, the recordings were kept in a locked cupboard; the data will be archived for 10 years. The local ethical committee approved the research project before it started.

## Findings

One theme emerged from the hermeneutical phenomenological analysis: *gaining useful life lessons through childhood experiences*. The participants’ experiences of events during childhood were illuminating the phenomenon of the inner child, understood as promoting or hindering health and well-being. The inner child became visible in the analysis of the participants experiences and can be described in the four sub-themes: S*haring relationships*, *playing to heal*, *being strong or frail* and *supporting the next generation.*

### Gaining useful life lessons through childhood experiences

The participants’ childhood experiences provided them with useful life lessons for health and well-being. The inner child became visible during their narrations when describing how they were sharing relationships, playing to heal and being strong or frail. In addition, the analyses revealed that these life lessons were not only useful for the adults during their life course, but also supporting the next generation of their own and/or other children. They describe how they make choices for their own health and well-being; for example, becoming stronger from tough experiences or copying the good experiences from childhood: “It’s a lot about relationships within the family, it’s not about doing things together all the time but caring for each other. Then I feel well” (A3). One participant said the following about bringing up their own children: “We are trying to teach our children and support them, to make them as well-educated as possible” (B4). Another, not yet a parent, said the following: “I have lived very protected and if I have children on my own I will give them security, even though I want them to experience life on their own” (B1). This is further described in the four sub-themes below.

#### Sharing relationships

The participants described the relationships they shared for good and for worse with parents, relatives and friends. By experiencing openness among peers and between generations the participants felt safe and secure. One participant explained: “In case of security for me, it has never been anything I couldn’t tell my parents” (A1). According to the participants, reliable relationships in a loving home taught them how to believe in themselves. These relationships developed confidence and freedom and became useful life lessons for the participants. They experienced loving actions in a number of ways including making sacrifices, supporting the child’s wishes and spending valuable time together. One participant stated: “I admire my mother who sent her only child abroad because she wanted a better life for me” (A4).

In addition, the participants experienced the importance of having close relationships with siblings and friends who accompanied them through their lifetime. It was also found that the participants were not always being treated well by others. School was one arena where the participants felt abandoned, ignored and bullied. One participant said: “I was about 10 years old, and I remember that they took my belongings and threw them around” (B9). The participants felt abandoned, lonely and not recognized in homes where parents were not in agreement, or were divorced or preoccupied with work.

#### Playing to heal

The participants described that the importance of reading and storytelling was a useful life lesson—not only for the development of thoughts, but also for the empathetic time that the parent and child could spend together. This created a base for experiences of awe and curiosity later in life. Participants’ experiences of reading at home varied due to the access of books at home. If there was a lack of books the problem was solved in a number of ways; one participant said: “My parents took us to the library, which I think was unusual for a family from the working class. They also read every evening before bedtime” (B5).

The participants experienced that play was a lot of fun and included football, table tennis and scouting. One participant remembered playing the same make-believe play for a long time: “It was almost our little secret. We didn’t dare to tell our friends that we still played this make-believe role play even when we were considered too old for this” (A2). The participants experienced that using their imagination and playing outdoors close to nature and animals with large spaces to play hide-and-seek were positive for mental health dimensions. The participants described the importance of athletics when growing up: “We had competitions at different athletic levels, and I got most of my friends through this interest” (B5). The participants not only increased the number of friends by participating in sports, they also increased their social competence—for example, prioritizing and making decisions. This knowledge and these skills were helpful in number of situations throughout life as useful life lessons.

The participants explained how they solved conflicts when playing with friends. Sometimes the play ended in wrestling or they just went home. The next day they were friends again. One participant explained: “I was a bit rigid, and playing helped me to become more flexible. It is a matter of give and take. It’s more fun playing together than on your own” (B6).

#### Being strong or frail

According to the participants, childhood experiences of being strong resulted in useful life lessons and the same was the case for the opposite polar of being frail. The participants described feeling strong, healthy and able to set barriers for things that they did not feel were right. Sometimes the participants became independent by spending time on their own together with grandparents: “There, at my grandparents’ house, I could go swimming or playing mini golf on my own” (A10). One participant felt that the parent trusted her ability to take responsibility. Even early in life, she had some moral feeling and could stand up against pressure from friends: “This meant that I never had the need for testing limits or revolting” (A7).

The participants described a drive or curiosity to go their own way and not be afraid of conflicts. Some participants mentioned being the first in the family to attend university, although they didn’t get much support from home. One participant explained: “Everybody else in my family had practical work like truck drivers. I became a civil engineer thanks to my independence and curiosity for things. I think that my parents thought that I studied too much” (B6). One participant felt the support from family but also her own strength which made it possible to get educated late in life: “I got educated through distance learning and had a great supporting network; I was also very motivated” (A9).

The participants experienced that being separated from their parents made them feel insecure and frail. The traumatic experiences were remembered through life and had an impact on the participants’ lives as adults. Situations like being left in a hospital as a child or being neglected by their parents could create separation anxiety as an adult in situations like moving away from home or breaking up with somebody. Not being able to ask for help made the situations worse, one participant said: “I remember when I stood up in my bed at the hospital crying for mom, I was four years old and felt abandoned. I still find separations difficult” (A5). Handling conflicts in relationships could cause feelings of being ill and frail. One participant narrated a childhood memory: “When I had a conflict I could not stand up for myself, and this affected me. Today at work I am not afraid of having an opinion and speaking up. I know that people listen and appreciate what I have to say” (A8). The participants shared other bad experiences that they managed to turn into something good. The participants exemplified how illness or a lack of recognition made them more empathetic, outgoing and understanding towards others as well as towards themselves. One participant said: “I became interested in music in my teens and today I am singing in choirs and playing theatre. This made me connect with people” (B7). The participants described various experiences connected to stress and burnout during life that was understood as them being frail. Stressful life situations were related to the strain of managing the life puzzle. One participant said: “I have to lower my ambition to be able to handle all pressures of everyday life” (A2). They experienced how stress got out of hand due to a heavy workload and a lack of social support. However, participants described alternative ways to handle stressful situations. One participant said: “Before I got burned out I quit my job” (B10).

#### Supporting the next generation

The participants described how positive and negative experiences from their childhood had become useful life lessons and could be used as an asset in their own role as a parent, caring adult to other children or in their work with children. The participants stated that they prioritized time spent together with their family, and they tried to teach their children that people are different and that you must compromise. One participant said: “I remember that we were outdoors in the nature with my grandpa and grandma, and I appreciated doing activities including all generations, even if it was not always enjoyable it was fun because everybody was there together” (B3). At the same time, the participants talked about the curiosity or the independence of doing things their own way and teaching their children to do the same. They found that their parents were good role models who protected them from the outside world. One participant said: “I want my children to feel secure about whom to turn to in case they need help from an adult” (A6). Participants with divorced parents described how important it was to take time off with their children, and especially that fathers spend time with their children despite having a stressful life. The participants wanted to support the next generation by emphasizing aspects of life they wanted but did not experience as children. One participant said: “This has had an impact on my life today. I believe it is important that my husband and his children spend time with each other and have a good relationship, even in everyday situations, which I didn’t always have” (A2).

Strong negative memories of disproportional injustice in their childhood could lead to extreme fairness towards their own children. Also, not being seen as a child and not getting the attention they longed for also became useful life lessons. One participant said: “It was more the things my parents didn’t do, that I think is what I want to do for my children. I want my children to have good values and respect others as well as themselves” (B2). The participants also told their children not to be afraid of making mistakes, and how this confidence could help them to see what possibilities they have in reaching certain goals in life. One participant felt that the freedom of choice was important: “The children must find their own future goals. What they find interesting I will support to 100%” (B8). The participants expressed the importance of being able to talk about anything with their parents or other adults. One participant expressed how the childhood experiences had become a useful life lesson: “I have decided that I don’t want to become like my mother and grandmother who didn’t talk about feelings. I think it is important to talk about what is meaningful in life and what is not” (A6). The participants described what they had learned through life and that they knew what was worth fighting for and what was better to leave.

## Discussion

A comprehensive understanding of the phenomenon of the inner child in relation to health and well-being as reflected in events during childhood experienced by adults showed that the inner child had a role in developing useful life lessons. The participants gained these life lessons through childhood experiences in accordance with Firman and Russel’s (1994) claim that the inner child is based on human beings’ life journey and has an impact on human lives. The findings highlight the importance of sharing relationships. Reliable relationships with parents, siblings and friends developed confidence and taught the participants how to believe in themselves, especially in situations when they felt abandoned, bullied and not recognized. This can be compared with Masten (2013) who argued that attachment relationships are important across the lifespan for adaptation. She highlighted that resilience, in recovery from disturbances, is a capacity for dynamic systems to continue developing in a healthy way. The participants’ experiences of being strong or frail impacted their health and well-being throughout life. They described a curiosity or strength to go their own way and not be afraid of conflicts. They also narrated about tough and challenging experiences like illness or a lack of recognition which they managed to turn into something good. The participants experienced tough times as a child and this could make them more understanding and empathetic towards themselves and others. Similarly, Werner and Smith (2001) showed examples of psychological resilience or ability, in spite of highly adverse conditions, successfully to adapt to life tasks. Masten (2013) discussed the possibility of resilience across generations—a parent and child in one generation shape the future capacity of the child as a parent in the next generation.

According to the participants, sports increased not only the number of friends, but also their social competence in how to make decisions and prioritize situations throughout life. Hertting and Kostenius (2012) found a positive connection between participation in organized leisure activities and health-promoting friendships, stress reduction and increased well-being in children. However, they emphasized that there is a need for a healthy balance related to the amount of activities—too many activities after school can increase stress and decrease well-being. The adults described only positive outcomes from leisure activities. This led us to believe that the participants kept a healthy balance in their activities or opted not to mention the negative aspects of leisure activities. In addition, the participants described how they were engaged in role-playing, make-believe play, reading and listening to stories which illuminate the presence of the inner child. These activities created a base for experiences of awe and curiosity later in life. Because the inner child has an impact on our lives as adults, the concept of play is important not only in childhood, but throughout the lifetime (Weston, 2009). According to Huizinga (1938/2004), play is an essential part of the development of human beings. In addition, the experiences of awe described by the participants can be compared with aspects of existential health highlighted by Melder (2011) as important for health-related quality of life.

The life lessons experienced by the participants in this study can be understood to show how fostering openness between generations increased trust and the feeling of security. These life lessons created motivation and an urge to support their own children and others to live a healthy life. According to Assagioli (1973) a sense of responsibility towards one’s family and society is a positive personal quality in adulthood. He went on to explain that the psychosynthesis of the ages is about keeping all ages alive within ourselves. That is, the best aspects of every stage of development in our past are synthesized. Similar elements can be found in the participants’ description of life lessons learned through negative as well as positive childhood experiences. This became a motivation to act responsibly and with care to support the next generation, which is similar to meaning and purpose in life described by Melder (2011) as aspects of the existential health dimension. In addition, the life lessons described by the participants in this study can be compared to the World Health Organization’s (WHO, 1998b) definition of health literacy—particularly the cognitive and social skills and ability of individuals to use information and understand in ways that maintain and promote good health. According to van der Heide et al. (2013), little knowledge is available today about health literacy among the general population in Europe, which is important as this contributes to more effective health promotion. They also shared findings from their own quantitative study in the Netherlands showing that being a male, having a low level of education or having low perceived social status were significantly related to low health literacy scores for accessing and understanding health information. Our qualitative study focused on the internal processes connected to health literacy and the phenomenon of the inner child. The study by van der Heide and colleagues discussed health information supplied by an outside source which the person needs to access, understand, appraise and apply. One can also view the information aspect of health literacy as an inner process based on childhood experiences. This is based on the understanding that all the past hidden ages have made up the person’s life journey, which Firman and Russel (1994) called the inner child. Kickbusch, Maag, and Saan (2005) argued that health literacy is active and dynamic, suggesting that society changes and that health-literate individuals are involved in continuous exchange and dialogue with the environment.

The findings highlight how adults are influenced by the inner child throughout their lifetime. This suggests that experiences during childhood impact how we adapt across the lifespan, and that attachment relationships are important within the family and outside the family, such as friends, teachers, partners and our own children. Comparisons can be made to Lamagna’s (2011) description of the “internal attachment system” (p. 284) where implicit memories associated with a person’s affects, thoughts, perceptions and behaviour govern his or her perceptions of the world and ways of being in it. The inner source of information, the presence of the inner child, is therefore worth considering in efforts to increase health literacy and especially in connection to the action competency aspect of health literacy described by Kickbusch et al. (2005). They explained that high health literacy skills offer a good ability to seek information and to take responsibility and make sound health decisions in the context of everyday life. In addition, they argued that health literacy skills are a critical empowerment strategy for human beings to increase control over their health. According to Mancuso (2011), overcoming health literacy barriers to integrate beliefs, values, social and cultural traditions with information related to health can be done based on an individual’s personal orientation of the world to make informed decisions and change health-related behaviour. This can be compared with Olander, Ringsberg, and Tillgren’s (2014) description of health literacy as a competence useful in everyday life to promote and maintain good health across a lifetime. Equally, health literacy as a competence useful in everyday life is found in the four sub-themes in the findings of this study: *Sharing relationships*, *playing to heal*, *being strong or frail* and *supporting the next generation.*

Even though stress and burnout were not the emphasis of this study, the participants described experiences of stress and burnout, especially when encountering the demands of life encompassing work, family and leisure, with the experience of not being in control or without social support. This is found in Eriksson’s (2016) description of adults today living in a stressful changeable world that creates challenges in coping with health and well-being. Despite stressful challenges, the participants experienced resilience as described by Masten (2013) as a capacity to find a healthy balance in life. It became evident how resilience was reiterated into the next generation in parent–child relationships based on the participants’ own childhood experiences.

### Methodological considerations

A weakness of this study might be that the number of participating adults was rather small. Although participants were from different educational, social and cultural backgrounds, no one described experiences of war or extraordinary traumatic situations. Therefore, there is a need also to include adults with these experiences to fully grasp the phenomenon of the inner child in relation to their health and well-being reflected in events during childhood. Kaplan (2006) offered a wider understanding of the value of play in creating distance from what is happening in the surroundings, as an opportunity to escape from reality, which was not described by the participants in this study. The somewhat homogeneous nature of the group of participants in connection to the lack of extraordinarily traumatic situations can therefore be viewed as a weakness in the study. According to van Manen (1990), pre-understanding might influence the findings in phenomenological research. To minimize this risk the authors discussed their own experiences and knowledge during the process of analysing the interviews. In phenomenological research the results can never be generalized, according to van Manen (1990), which is a limitation in this study. However, the results from this study can contribute to increasing the understanding and knowledge about how adults’ health and well-being relate to the phenomenon of the inner child.

### Conclusions and final thoughts

The findings indicate that the participants learned useful life lessons suggesting that experiences during childhood impact how we adapt across the lifespan, and that attachment relationships are important within the family and outside the family, such as with friends, teachers, partners and our own children. However, the findings from this qualitative study need to be further examined in connection to health literacy practice. We would therefore suggest using the following conclusions and final thoughts to inform future research.

In line with Firman Russel (1994), the findings suggest that the inner child has a role in developing useful life lessons that the participants gained through childhood experiences. These life lessons created motivation to support their own children and other young people to live a healthy life. The following surfaced as useful life lessons for the participating adults, illuminating the presence of the inner child:

- Reliable relationships with parents, siblings and friends helped instil confidence and taught the participants how to believe in themselves, involving social dimensions of health and well-being.
- Challenging times as a child made them more understanding and empathetic towards themselves and others, involving mental and social dimensions of health.
- Engaging in role-playing, make-believe play, reading and listening to stories showed the presence of the inner child, and this created a base for experiences of awe and curiosity later in life, involving existential dimensions of health and well-being.
- Openness between generations increased trust and the feelings of security, involving mental, social and existential dimensions of health and well-being.

In health literacy efforts, the health information aspect can be viewed as an outside source, verbal and written sources, i.e., brochures about health, a health educator’s verbal information about health promotion practice, and also an inner process in which the person renegotiates childhood experiences to make them useful in the promotion of health and well-being. In other words, recognizing the inner child’s presence and its effects on a person’s health and well-being can be an asset when aiming to increase health literacy. Barry (2009) stressed the importance of supportive environments and the role of schools, workplaces and communities as key contexts of settings for promoting positive mental health. With these contexts in mind we suggest including childhood experiences in dialogues when aiming at promoting positive mental health. One example is the questions we posed in this study—*Please describe significant events from your childhood that you have carried with you throughout life Is there anything in what you have narrated that has affected your health and how you feel today?*

The findings of this study can be a contribution to the health literacy discussion and detail how knowledge and action competency is developed in mental, social and existential dimensions of health and well-being We argue that this knowledge about the phenomenon of the inner child in relation to health and well-being can be useful in health promotion efforts to increase health literacy—an area in need of further research.
